# Supplementary material for: Peptides Regulating Proliferative Activity and Inflammatory Pathways in the Monocyte/Macrophage THP-1 Cell Line
Source: Int J Mol Sci. 2022 Mar 25;23(7):3607. doi: 10.3390/ijms23073607 (PMC8999041; doi:10.3390/ijms23073607)
Supplement: Supplementary file 1 [file ijms-23-03607-s001.zip › ijms-1615048-supplementary.pdf]

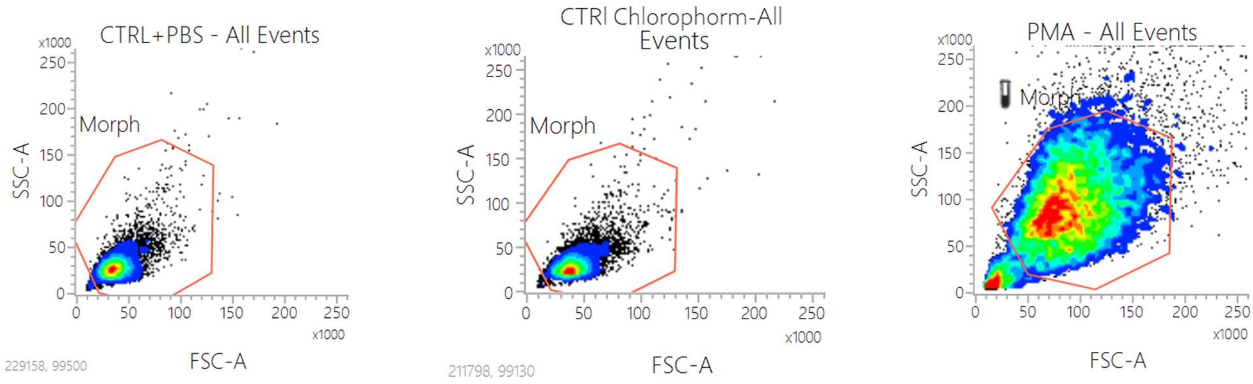

Statistics

Name	FSC-A Mean	SSC-A Mean	Events/ul
**PMA:Morph**	97,808	94,250	347.04
**CTRL+PBS: Morph**	41,004	27,651	3,299.67
**CTRL Chlorophorm: Morph**	43,735	27,237	4,951.00

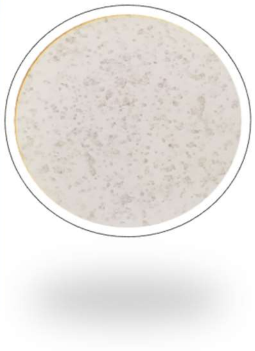

***Supplementary Figure* S1.** Cytometry analysis of monocytes/macrophages shift by PMA. Cytofluorimetric profile of THP-1 cells upon treatment with the PMA or the vehicle alone (Chlorophorm). The granulocytic cytoplasm density and shape modulation is documented through light scattered transmission. (Morph. indicates granulocytic size and conformation of scattered cells)

THP-1 COUNT CELLS

**C**	300,000
**P1**	1,000,000
**P2**	800,000
**P3**	1,600,000
**P4**	700,000
**P5**	750,000

Plated 200,000 in 10 mL of RPMI medium.

A Counted after 16 hours of treatment with each peptide.

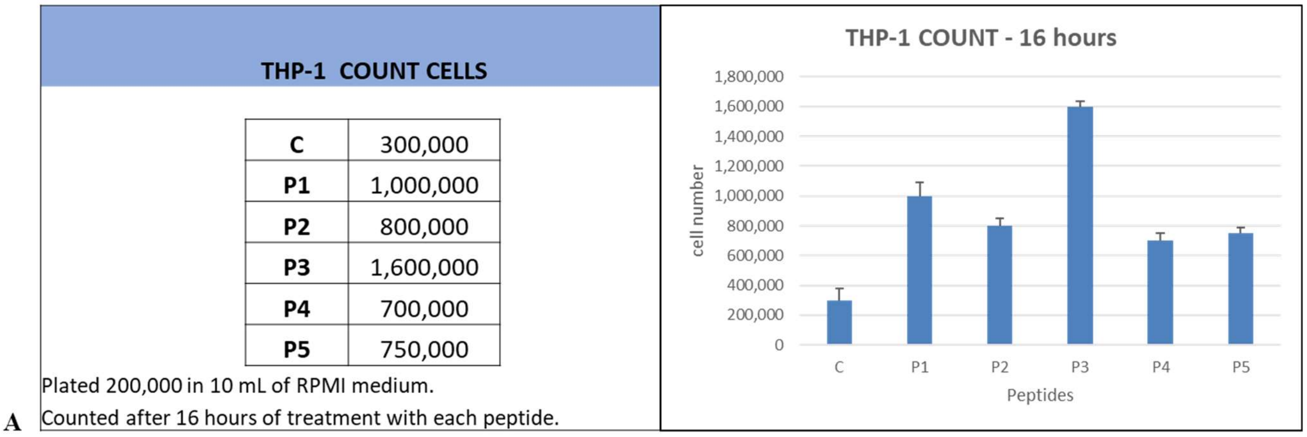

Supplementary Figure S2 A. Cell Growth Assay. 2 x 10^5 cells were plated into 10 ml RPMI medium on dish plates. Viable cells were counted after ;16 hrs. incubation using the trypan blue exclusion method.

B

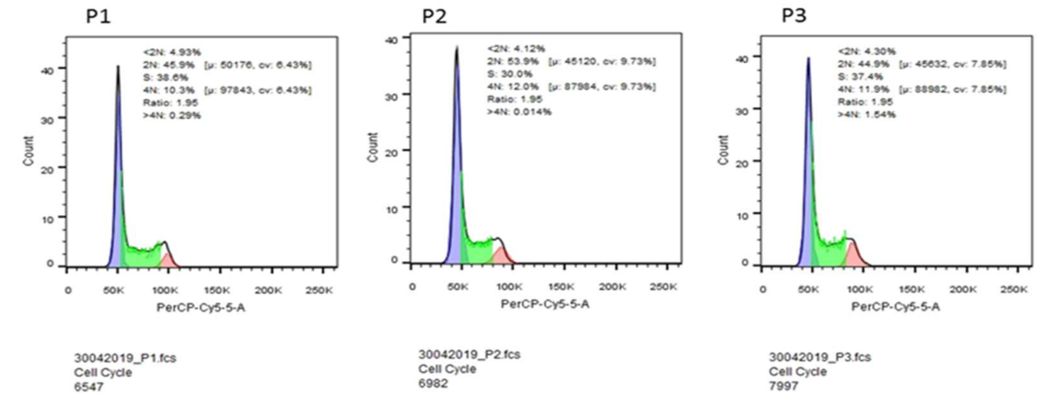

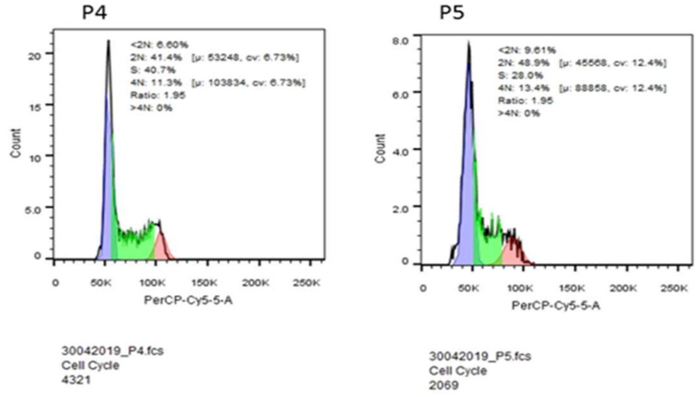

%	G1	S	G2	Apoptosis
Ctrl	39	42.15	17	1.98
P1	45.9	38.6	10.3	3.98
P2	53.9	30	12	3.79
P3	44.9	37.4	11.9	2.1
P4	41.4	40.7	11.3	5.92
P5	48.9	28	12.9	9.73

Supplementary Figure S2 B. Dynamic Function of cell cycle. Phase modulation after peptides induction, measured by flow cytometry. Greater apoptosis was present in monocytes only after induction due to Biopeptide Chonluten (P5). Numbers are expressed as percentage of cells.

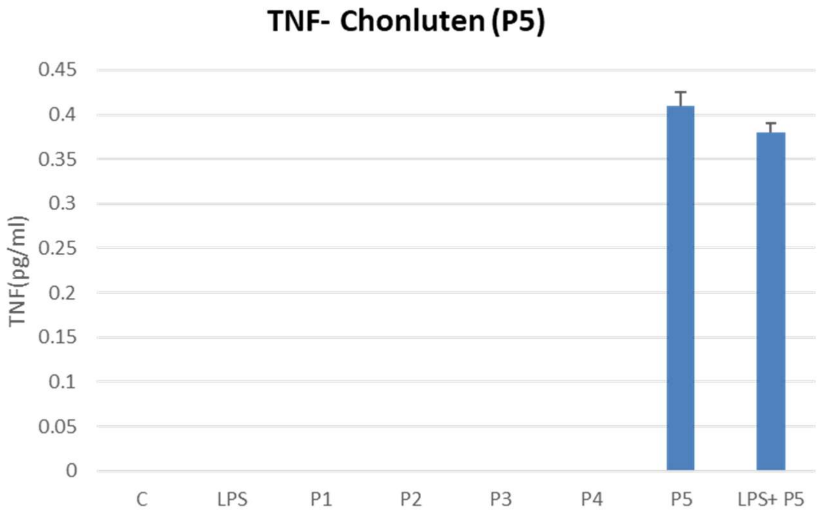

Supplementary Figure S3. TNF modulation on THP-1 Monocyte cell line. TNF-a was measured according to cytometry assay. Data were from at least three different sets of experiments. Monocytes were treated as indicated.

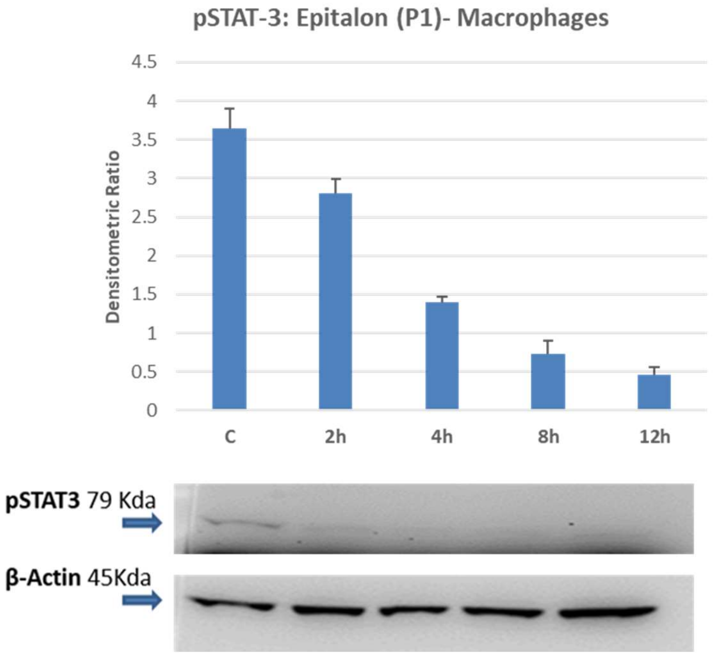

Supplementary Figure S4: pSTAT3 modulation by Epitalon (P1) peptide. Slightly decrease of pSTAT3 phosphorylation of THP-1 macrophages after time course treatment with Epitalon (P1). Densitometric analysis was performed normalizing band intensity to β-actin expression
